# Development and Synthesis of Bombesin-Based Radiopharmaceutical Precursors Modified with Knottin

**DOI:** 10.17691/stm2024.16.2.01

**Published:** 2024-04-27

**Authors:** E.A. Beloborodov, E.V. Iurova, A.N. Fomin, Yu.V. Saenko

**Affiliations:** Researcher, Laboratory for Peptide Drugs and Vaccines Development, S.P. Kapitsa Research Institute of Technology; Ulyanovsk State University, 42 Leo Tolstoy St., Ulyanovsk, 432017, Russia; Junior Researcher, Laboratory for Peptide Drugs and Vaccine Development, S.P. Kapitsa Research Institute of Technology; Ulyanovsk State University, 42 Leo Tolstoy St., Ulyanovsk, 432017, Russia; PhD, Senior Researcher, Laboratory for Peptide Drugs and Vaccines Development, S.P. Kapitsa Research Institute of Technology; Ulyanovsk State University, 42 Leo Tolstoy St., Ulyanovsk, 432017, Russia; DSc, Leading Researcher, Laboratory for Peptide Drugs and Vaccines Development, S.P. Kapitsa Research Institute of Technology; Ulyanovsk State University, 42 Leo Tolstoy St., Ulyanovsk, 432017, Russia

**Keywords:** bombesin, knottin, targeted therapy, peptide toxin, bombesin receptor

## Abstract

Bombesin receptors on the cell surface are of great interest as a target for targeted cancer therapy. One of the strategies of targeting bombesin receptors involves the use of tropic short peptides. However, the main limitation for the wide application of peptides as drugs is their low stability *in vivo* due to their sensitivity to extreme conditions of the internal body environment such as temperature and action of enzymes. In our work, a short bombesin peptide, taken as a basis, was modified with a knottin, a toxin with an inhibitor cystine knot, increasing thereby the stability of the short peptide under various conditions.

**The aim of the investigation** is to study the chemical and radiochemical stability of the structure based on the short bombesin peptide and knottin, as well as the ability of the obtained structure to bind to tumor cells.

**Materials and Methods:**

The work analyzed the chemical and radiochemical stability of the synthesized peptide labeled with a lutetium radioisotope using high-performance liquid chromatography. A fluorescent-labeled peptide, obtained by a solid-phase peptide synthesis, was used to analyze binding to cultures expressing bombesin receptors.

**Results:**

The analysis has shown increased chemical and radiochemical stability of the knottin-modified peptide, as compared to the commercial analog, and maintenance of a high ability to bind to receptors on the surface of cancer cells.

**Conclusion:**

The structure created on the basis of a short bombesin peptide and knottin possesses increased stability and retains the ability to bind to cancer cells. All this allows us to consider the creation of these structures as a strategy for fabricating stabilizing scaffolds for short peptides for a peptide-receptor therapy.

## Introduction

Peptide receptor radionuclide therapy represents a molecular-targeting radiotherapy including systemic administration of radiolabeled peptide aimed to influence the receptors on the surface of the tumor cells [1]. The largest class of receptors, which over-express in oncological diseases, are G protein-coupled receptors (gastrin-releasing peptide receptors, GRPR). This class includes over 800 receptors having a common structure of seven transmembrane helices, which are coupled by three intracellular and three extracellular loop domains, extracellular N-terminal, and intracellular carboxyl-terminal domain [2]. A family of bombesin receptors is also referred to the GRPR class [3]. Bombesin receptors are widely distributed in mammalian organisms. Their activation evokes a wide spectrum of physiological reactions: stimulation of normal and neoplastic tissue growth, contraction of the smooth muscles, eating behavior, secretion, and many effects of the central nervous system including regulation of the circadian rhythm [4, 5]. However, the bombesin receptors attracted attention of the researchers primarily due to their expression in various types of cancer [6]. They are often express in great amount on the tumor cells of the mammary gland, prostate gland, cancer of the colon, lungs, neuroblastoma, and other cells [7–13]. In this connection, peptide-receptor therapy, directed to the bombesin receptors, may serve as a promising base for treating a wide spectrum of oncological diseases.

Application of tropic short peptides underlies one of the strategies aimed at targeting bombesin receptors. Amidated tetradecapeptide, called bombesin, isolated from the skin of *Bombina bombina* frog still in 1972 by Erspamer et al. [14], may be referred to these peptides. Later bombesin-like peptides were found in mammals as well. Bombesin-like peptide 1, BB1 (decapeptide neuromedin B, NMB), encountered in the precursor forms of 30 and 32 amino acids, was isolated from porcine spinal cord. The terminal carboxyl NMB groups are identical to bombesin excluding the groups of replacing valine with threonine in NMB [15]. Bombesin-like peptide 2, BB2 (gastrin-releasing peptide, GRP), of 27 amino acids was isolated from the porcine stomach. It had 7 carboxyl terminal amino acids identical to bombesin, which provide similar biological activity [16]. Finally bombesin-like receptor 3, BRS3, also known as bombesin receptor subtype 3 has been discovered, but presently it has been weakly studied and its natural ligand has not been identified [17].

In our work, we created a new non-natural peptide construct based on the knottin toxin capable of target bonding to the bombesin receptor on the surface of cancer cells. A short peptide bombesin isolated from the skin of the *B. bombina* frog was taken as the basis. It was modified using a toxin with an inhibitor cystine knot. This class of toxins includes primarily the toxins from invertebrate venom, which are known to possess a high proteolytic and thermal stability. We have chosen the knottin toxin U5-scytotoxin-Sth1a (UniProt: U51A_ SCYTH) derived from the spider *Scytodes thoracica*. The chosen short peptide was placed in the position between the first and second cysteine residues and a new created structure was called BBN/C1-C2. In our previous investigation [18], when we worked with the peptide showing tropism to prostate-specific membrane antigen (PSMA), we have demonstrated the effectiveness of this strategy.

Additionally to the peptides, an important task in the peptide-receptor radionuclide therapy is to select a radioisotope whose decay activity is detected during tumor imaging and provides elimination of tumor cells in case of treatment. One of the promising isotopes for the application in the radiopharmaceuticals is lutetium. It has a short half-life period of about 7 days, which is long enough for transport and storage, but sufficiently short to protect the organism against accumulation of high radiation doses. Lutetium is a beta- and gamma-emitter, that allows using it both for therapy and imaging. The energy of beta-particles provides ideal penetration into soft tissues to the depth of 670 μm, being sufficient for the destruction of a tumor cell but insufficient for producing a negative effect on the surrounding tissues [19].

The chelator, included in the radiopharmaceutical and providing coupling of the radioisotope with the peptide construct, plays an important role in the peptide-receptor radionuclide therapy. We used lutetium (^177^Lu) chloride as a radioisotope in our work. It has the degree of oxidation of +3, facilitating ion chelation, and was considered to be the best candidate for our case. Bifunctional microcyclic DOTA (2,2′,2′′,2′′′-(1,4,7,10-Tetraazacyclododecane-1,4,7,10- tetrayl)tetraacetic acid) was chosen as a chelator. Similar chelators are capable of forming highly stable metal complexes with ions of trivalent metals (Lu^3+^, Ga^3+^), which determines their use in the development of radiopharmaceuticals [20, 21].

Thus, creating a new radiopharmaceutical, it should be taken into consideration not only the degree of its binding to the receptors on the surface of the tumor cells but stability as well, which shows itself in the ability of the peptide construct to remain constant (chemical stability) and to retain a radioisotope (radiochemical stability).

**The aim of the investigation** is to study the chemical and radiochemical stability of the structure based on a short bombesin peptide and knottin toxin, BBN/C1-C2, as well as the ability of the obtained structure to bind to tumor cells.

## Materials and Methods

### Synthesis and analysis of the BBN/C1-C2 peptide construct

Modification and synthesis of bombesin was carried out in peptide synthesizer ResPep SL (Intavis, Germany) using Fmoc-protected amino acids on the TentaGel resin (Intavis, Germany). Synthesis was started with deprotection of amino acids with 20% 4-methylpiperidyne (Acros Organics, Belgium) in dimethylformamide (Chemical Line, Russia). Next, amino acids were activated with HBTU (hexafluorophosphate benzotriazole tetramethyl uronium) (Chemical Line, Russia) in the presence of N-methyl-2-pyrrolidone and N-methylmorpholine (PanReac AppliChem, Spain).

Binding was performed using 5% acetic anhydride (Sigma Aldrich, USA). During synthesis, dimethylformamide was the main solvent. Removal from the resin was done with a mixture of 92.5% trifluoroacetic acid (PanReac AppliChem, Spain), 5% triisopropylsilane (Acros Organics, Belgium), and 2.5% deionized water for 3 h at room temperature. The peptide was then precipitated by centrifugation using glacial tert-butyl methyl ether (Chemical Line, Russia). The DOTA chelator was used as a chelating agent for the radioisotope. It was attached in the process of the peptide synthesis when it was added in the form of tris-DOTA (Intavis, Germany).

PSMA-617 (Huayi Isotopes, China), targeting PSMA, was employed for the comparison due to the absence of commercial preparations targeting the bombesin receptors.

The analysis of the synthesis and purity control of the commercial PSMA-617 (Huayi Isotopes, China) was performed using chromatographic system Shimadzu LC-20AD XR (Shimadzu, Japan), equipped with spectrophotometric detector SPD-20A, according to the principle of the reverse-phase chromatography with the help of Luna C18 column (Dr. Maisch, Germany) and gradient elution of acetonitrile (Criochrome, Russia) from 95 to 0% and deionized water from 0 to 100% for 40 min. Mass-spectrometric analysis was conducted on the basis of MALDI-TOF mass-spectrometry using MALDI-TOF MS system, series FLEX (Bruker Daltonics, Germany). The peptide was purified by means of a high-performance liquid chromatography system NGC Quest 10 (Bio-Rad, USA) using Bio-Gel P-4 sorbent on the Econo-Column 1×30 cm (Bio-Rad, USA).

### Labeling of the peptide construction BBN/C1-C2 and PSMA-617 with lutetium isotope and stability analysis

Lutetium (^177^Lu) chloride was used as a radioligand. BBN/C1-C2 and PSMA-617 were labeled at 80°C for 20 min at 1:10 mole ratio of lutetium chloride (100 MBq in 0.05 N chloric acid) and the peptide in the acetate buffer with pH 4 in the presence of ascorbic acid. The radioactivity of samples was measured with a gamma counter ISOMED 2010 (PTW, Germany). Labeling of the peptides was conducted on a Gaia/Luna complex (Elysia-Raytest, Germany). Radiochemical purity was analyzed on the Shimadzu chromatograph (Shimadzu, Japan) with a GABI detector (Elysia-Raytest, Germany) at acetonitrile/water gradient: 0–5 min — 2% of acetonitrile, 5‒20 min — 95% of acetonitrile, 20–25 min — 2% of acetonitrile; water was acidified with trifluoroacetic acid (0.01%). The system was controlled by the Clarity programs (Clarity Software, Great Britain). The labeled peptides were purified from free lutetium with the help of single-use cartridges C18 (ABX, Germany), the GAIA complex was used according to the gel-filtration principle. Radiochemical stability was evaluated using the same parameters after 24, 48, 96 and 168 h in 0.9% sodium chloride at 37°C. Chemical stability was similarly fixed in 0.9% sodium chloride as described above. Chromatographic purity was calculated as a percentage of the target substance peak area in relation to the total area of the peaks on the chromatogram taken as 100%.

### Labeling of the peptide construct BBN/C1-C2 and PSMA-617 with a fluorescent dye

A fluorescent dye 6-FAM in the form of amine (Lumiprob, Russia) was used for bonding analysis. The peptides were labeled according a standard manufacturer protocol: they were dissolved in 0.1 mol/L solution of NaHCO3, 6-FAM dye was then added to the solution and incubated for 30 min at room temperature in the dark. The peptides were purified from the dye excess using alcohol precipitation method. For purification, one part of the labeled peptide solution was mixed with nine parts of the 95% ethanol cooled at –20°C. Further, the peptides were incubated for 90 min at the same temperature (–20°C). After incubation, the cooled solution was centrifuged for 15 min at 13,000 rpm in the Allegra centrifuge (Beckman Coulter, USA). After centrifugation, the supernatant, containing the labeled peptide, was removed and dried using a vacuum concentrator (Eppendorf, Germany).

### Analysis of 6-FAM-labeled peptide bonding to the cell cultures

The peptides were bound to the cell cultures such as HCT-116, LnCap, PC-3, CHO-K1. The HCT-116 (human colorectal carcinoma cell line) and CHO-K1 (an epithelial cell line derived from the Chinese hamster ovary) were obtained from the Russian collection of cell cultures of vertebrate. The LnCap (PSMA-positive adenocarcinoma of the human prostate gland) and PC-3 cultures (PSMA-negative adenocarcinoma of the human prostate obtained from the bone metastases) were kindly provided by the N.N. Blokhin National Medical Research Center of Oncology (Russia). The HCT-116 and PC-3 cultures were kept on the RPMI-1640 culture medium (PanEco, Russia) with the addition of 10% fetal bovine serum (Biosera, France) and gentamycin at the final concentration of 40 μg/ml. The LnCap culture was kept in RPMI-1640 medium (PanEco, Russia) supplemented with 10% fetal bovine serum (Biosera, France), sodium pyruvate, vitamins for RPMI, and penicillin/streptomycin (PanEco, Russia). The CHO-K1 culture was kept in DMEM/F12 medium (PanEco, Russia) supplemented with 10% fetal bovine serum (Biosera, France) and gentamicin at the final concentration of 40 μg/ml. All cultures were held in the CO_2_-incubator (Sanyo, Japan) at 37°C and 5% CO_2_, passages were performed every 3‒4 days using 0.25% trypsin (PanEco, Russia), the experiment was carried out within the first three passages.

24 h before the experiment, the cultures were seeded in 24-well plates at the concentration of 100,000/well (LnCap — 150,000/well) to achieve the exponential stage. Next, the FAM-labeled peptides BBN/C1-C2 and PSMA-617 were added to the culture at a final concentration of 1 μmol/L and incubated for 3 h. After the incubation, the medium with unbonded peptides was removed, the plate wells were washed with a cooled phosphate buffer and the fluorescence signal was recorded using an optical system equipped with a Nikon Ti-S microscope, DS-Qi1MC camera, S Plan Fluor ELWD 20×0.45 objective lenses, 465-495/515-555 nm filter (Nikon, Japan), and a computer with NIS-elements 4.0 software package.

The quantitative analysis of images was performed with an ImageJ program. The fluorescence signal from more than 300 cells was analyzed for each peptide. The fluorescence intensity was calculated by the following formula: total cell fluorescence = integral density – (area of the selected cell × mean fluorescence of the background indicators) as described by Khokhlova et al. [22]. Shortly it looks like as follows: to obtain a fluorescence signal of each selected cell in the ImageJ program, the integral density parameter is taken, after that, the fluorescence of the background, equal in area to the cell size, is subtracted from this parameter. The fluorescence signal without peptides was registered as a control. The data were presented in the relative units of fluorescence.

### Expression analysis of genes encoding the target receptors

The total RNA was isolated from the HCT-116, LnCap, PC-3, and CHO-K1 cell lines using Proba-NK kit (DNA-Technology, Russia) following the instruction manual, kDNA was synthetized by means of the MMLV RT kit (Eurogen, Russia) for reverse transcription with random decanucleatide primers. PCR with the detection in real time was conducted using a 5X qPCRmix-HS ready-made mixture for PCR with Taq-polymerase (Eurogene, Russia) on the CFX96 amplifier (Bio-Rad, USA). To determine a relative expression level for *GRPR* and *FOLH-1* genes (PSMA), results were equalized on mRNA of the housekeeping gene *GAPDH*. Reaction by *GAPDH* gene served also as an internal control for determining the efficacy of the RNA isolation and the process of reverse transcription. In order to perform PCR in real time, species-specific primers and probes were selected on mRNA of *GRPR*, *FOLH-1* (PSMA) genes and human *GAPDH* and *Cricetulus griseus GAPDH* (when analyzing RNA isolated from the CHO-K1 cell line). The primers were chosen according to the exon-exon junctions for obtaining specificity in relation to mRNA, absence of the positive result on the genome DNA was tested by performing PCR without reverse transcription on the specimens of human genome DNA and also DNA isolated from the CHO-K1 cell line. The expression level was calculated by the following formula:

R=2[CtGAPDH-Ct(GRPR/FOLH-1)],

where *R* is a relative expression level of *GRPR*/*FOLH-1* gene; *Ct*GAPDH — average threshold cycle (*Ct*) of *GAPDH* reaction; *Ct*(GRPR/FOLH-1) — average threshold cycle (*Ct*) of *GRPR*/*FOLH-1* reaction.

### Ethical considerations

No humans or animals were directly involved in the process of biomaterial selection during this investigation. The Russian laws and regulations do not require approval for the usage of the cell line biomaterials for scientific researches (Federal Law of June 23, 2016 No.180-FZ).

### Statistical data processing

Binding analysis data were processed using OriginPro software (OriginLab Corporation, USA; v. b9.5.0.193) and the Student’s t-test, Mann–Whitney test was applied to process data of the stability analysis. To determine the character of distribution, we used the asymmetry and excess criterion. Since the data of the binding analysis were compared to the control group and also with the negative group (CHO-K1 culture), the Bonferroni test was used to eliminate the effect of multiple comparisons and differences between the groups were considered statistically significant at p≤0.02. All data were presented as M±SD, where M is mean, SD — standard deviation.

## Results

### Synthesis and analysis of peptides

**Figure 1.**
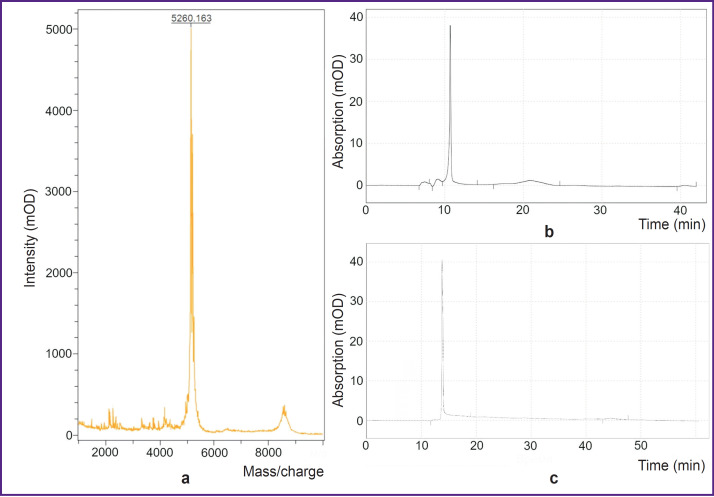
Mass-spectogram (a), chromatogram (b) of the peptide construct BBN/C1-C2, and (c) chromatogram of PSMA-617

A peptide construct BBN/C1-C2 with a molecular mass of 5266.935 Da and purity over 80% was obtained as a result of solid-phase synthesis (Figure 1).

As the molecular construct was created on the basis of knottin, which forms three disulphide bridges, refolding of disulphide bonds was necessary. As a result, the molecular mass of the construct changed to 5260.163 Da. After the subsequent purification, purity of the folded peptide was 95.1% as compared to 99.1% for the commercial PSMA-617.

### Labeling of the peptide construct BBN/C1-C2 and PSMA-617 with lutetium isotope and stability analysis

**Figure 2.**
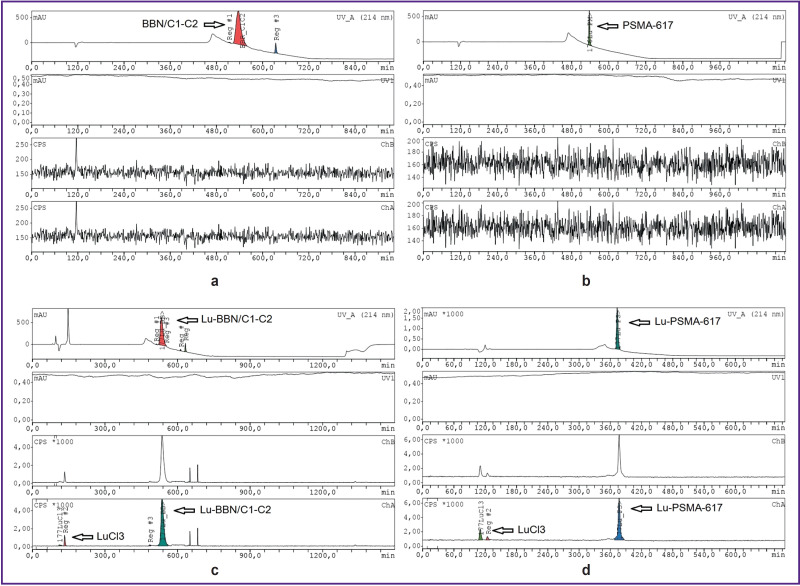
Radiochromograms of BBN/C1-C2 peptide construct (a), (c) and PSMA-617 precursor (b), (d) before labeling (a), (b) and after labeling (c), (d)

**Figure 3.**
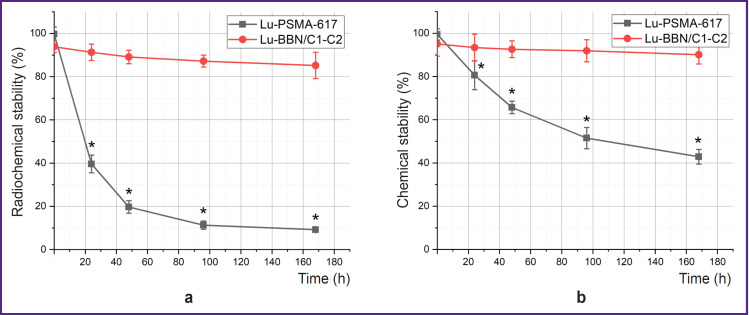
Radiochemical (a) and chemical (b) stability of BBN/C1-C2 and PSMA-617 * statistically significant difference with BBN/C1-C2

The labeling yield of 80–90% was achieved in the process of labeling BBN/C1-C2 and PSMA-617 with lutetium ^177^Lu (Figure 2). The stability analysis of the labeled structures has shown that radioisotope detachment from Lu-PSMA-617 occurred already in the first 24 h, radiochemical purity decreases from (99.7±3.2) to (39.6±4.1)% (Figure 3 (a)).

48 h after labeling, radiochemical purity of Lu-PSMA-617 reduces to (19.7±2.9)%, and 168 h later it becomes (9.2±0.7)%. Chemical stability of PSMA-617 also decreases. So, in the first 24 h, the purity drops from (99.1±2.6) to (80.6±6.7)%, after 168 h it makes up (42.9±3.4)% (Figure 3 (b)). At the same time, the knottinbased BBN/C1-C2 preserves not only radiochemical stability but chemical as well. The chemical stability of BBN/C1-C2 after 24 h changes from the initial (95.1±5.7) to (93.4±6.2)%, after 48 h to (92.6±3.9)%, and after 168 h it reduces to (90.1±4.3)% (see Figure 3 (b)). The radiochemical purity also reduces insignificantly: 24 h after labeling it becomes (91.3±3.8)% compared to the initial (93.8±2.6)%, after 48 h — (89.1±3.1)%, after 168 h — (85.2±6.2)%.

**Figure 4.**
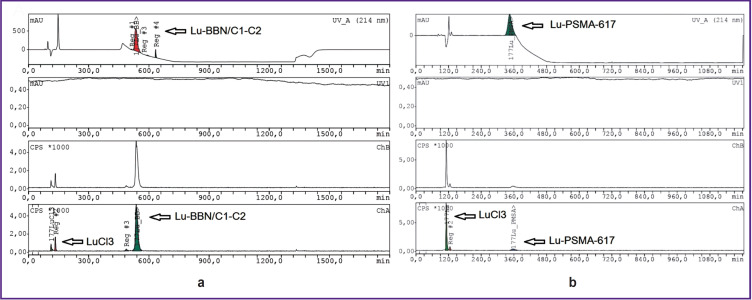
Lu-BBN/C1-C2 (a) and Lu-PSMA-617 (b) radiochromatograms

Radiochromatograms of the labeled structures after 168 h are presented in Figure 4, which demonstrates that after 168 h the radioisotope has almost completely detached from the PSMA-617 molecule, while BBN/C1- C2 maintains the labeling result at the level of 80%.

### Analysis of binding 6-FAM-labeled peptides to the cell cultures

**Figure 5.**
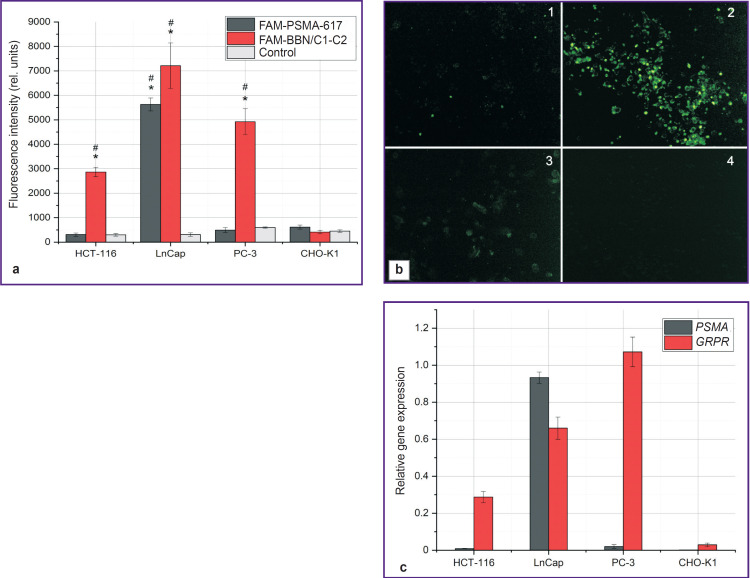
Results of binding 6-FAM-BBN/C1-C2 and 6-FAMPSMA- 617 (a) to the cultures HCT-116 (b, 1), LnCap (b, 2), PC-3 (b, 3), and CHO-K1 (b, 4) according to the expression level of the gene encoding target receptor (c) (photo illustration for 6-FAM-BBN/C1-C2 is presented) * statistically significant difference from the control, ^#^ statistically significant difference from the CHO-K1 culture

Since not only chemical and radiochemical stability is the decisive factor for target structures but their ability to attach to the receptors on the surface of the cancer cells as well, we analyzed binding of fluorescent-labeled molecules of BBN/C1-C2 and PSMA-617 with the appropriate receptors on the cell surface, i.e. bombesin receptor for the BBN/C1-C2 molecule and prostate-specific membrane antigen for the PSMA-617 (Figure 5). It can be seen that the PSMA- 617 molecule is binding to the LnCap culture as these cells over-express PSMA (Figure 5 (c)). At the same time, this molecule is not bound to HCT-116, PC-3, and CHO-K1 cultures due to the absence or low expression of the target receptors. On the contrary, the BBN/C1-C2 peptide structure based on the tropic short bombesin peptide and knottin binds to a considerable degree to all three tumor cultures, especially to the LnCap, since these cultures over-express the bombesin receptor, while the CHO-K1 culture expresses this receptor in the insignificant quantity and, therefore, the fluorescence signal was not registered during the experiment.

## Discussion

One of the key aspects of cancer therapy is a target delivery of therapeutic agents to cancer cells to make treatment maximally effective and to minimize damage to healthy tissues. Bioactive peptides attracted attention owing to their potential anticancer properties. Approaches based on the application of peptides offer some advantages in cancer treatment including high specificity, reduced normal tissues toxicity, and versatility of acting on various molecular ways involved in cancer progressing [23]. For example, the first generation of bombesin radioligands were presented by GRPR agonists derived from the C-terminal fragments of amphibian bombesin tetradecapeptide. GRPR agonists possess the property of internalization when binding to receptors, which facilitates long-term retention of the preparation in the target cell and is considered a prerequisite for high absorption *in vivo* [24]. One of the first radioligand of bombesin receptor was (99m)Tc-N(3)S-X-BBN[7-14]NH(2) preparation [25]. The preparation was tested on a small group of patients with breast and prostate cancer and helped visualize four of six cases of breast cancer and one of four androgen-resistant prostate carcinomas metastasizing to the bones [26]. One more investigation [27] has shown Ga-labeled GRPR antagonist as a promising agent for cancer imaging. The work was carried out on the model of PC-3 tumor cell xenograft. The examined GRPR antagonist demonstrated a high absorption by the tumor at extremely low absorption by healthy tissues, which gives a high tumor-to-background tissue contrast during PET-examination.

The main limitation for a wide application of peptides as drugs is their low stability *in vivo* due to the sensitivity to the extreme conditions of the internal body environment such as temperature and enzyme action [28]. One of the key proteolytic enzymes is neutral endopeptidase responsible for catalytic inactivation of many neuropeptide substrates including bombesin-like peptides. Neutral endopeptidase expresses in abundance in the human body including vascular walls, the main organs and tissues, making imaging and therapy potentially difficult by breaking down radiopeptides on the amino acid side chain of hydrophobic amino acids. The released radiometabolites cannot interact with target receptors associated with the tumor and, as a result, the diagnostic sensitivity and therapeutic efficacy are seriously reduced [29]. Another problem of therapeutic peptide application is their fast renal clearance connected with the sensitivity of peptides to filtration by renal glomeruli due to their small dimension and hampered re-adsorption through renal canaliculi [30]. Enlargement of physical molecular size will promote renal clearance resistance and increased time of molecule circulation in blood.

One of the ways to overcome the limitations in relation to proteases and to increase of peptide stability to the body’s internal environment is the application of peptides with a cystine knot. These peptides are referred to the class of peptides of 30–50 amino acids long. Owing to their specific 3D structure, they possess high stability to increased temperature and protease effect. In our work, we used a short bombesin peptide stabilized by the inclusion of U5-scytotoxin-Sth1a toxin, derived from the *Scytodes thoracica* spider, into the structure. The comparative analysis of BBN/C1-C2 with PSMA-617 was carried out in view of the absence of radiopharmaceuticals targeting bombesin receptors.

Presently, similar investigations using knottins as a scaffold for therapeutic peptides are being actively carried out. For example, Silverman et al. [31] used a shorten form of agouti-related protein (AgRP), the peptide with a cystine knot as a molecular scaffold for the peptides with tropism to α_v_β_3_-integrins. The obtained synthetic peptides have shown a higher specificity to the target receptor and absence of non-specific binding typical for the initial therapeutic peptide. However, these constructed peptides, as described above, possess lower stability than knottin-based peptides derived from natural sources.

In 2021, Kimura [32] published his work, in which he studied the possibility to use knottin GTx1-15, isolated from *Grammostola rosea* bird spider, as a scaffold for therapeutic peptides. Knottin has demonstrated high stability in blood plasma, low cytotoxicity, and antigenicity. This knottin is considered by the author as an exceptionally promising scaffold for future therapeutic molecules. However, no further investigations with practical application of GTx1-15 knottin have been conducted.

The BBN/C1-C2 structure, in comparison with PSMA-617, maintains both chemical and radiochemical stability for a long time (see Figures 3 and 4). For example, BBN/C1-C2 has the chemical purity of (90.1±4.3)% and the radiochemical purity of (85.2±6.1)% after 168 h. At the same time, PSMA-617 preserves the chemical purity (42.9±3.4)% and radiochemical one (9.2±0.7)% after the same period of time.

It was separately considered whether incorporation of tropic peptide into the toxin structure influences binding to the surface of cell cultures (Figure 5 (a)). PSMA-617 showed a high degree of attachment to the LnCaP culture, which is connected with high expression on the surface of prostate-specific membrane antigen cells targeted by this preparation (see Figure 5 (c)). In case of the BBN/C1-C2 construct, the peptide bound both to the HCT-116 and to the LnCap and PC-3 cultures. All these cultures express bombesin receptors on their surface [33–35]. However, no fluorescence signal was recorded during our experiment, when we analyzed binding of both peptides to the CHO-K1 culture, which was due to the absence of the PSMA receptor expression or to a weak expression of GRPR receptor. In this case, CHO-K1 was used as a control culture to validate the absence of competitive binding of the BBN/C1-C2 structure to other cell receptors and ion channels. The BBN/C1-C2 molecule was created on the basis of the toxin able to attach to the mammalian ion channels, and in this regard, there was a possibility that the examined molecule would preserve the ability to bind to ion channels on the surface of the normal cells irrespective of the presence of the incorporated short peptide [36]. However, our study has shown that the BBN/C1-C2 molecule binds only to the target bombesin receptors, which is most likely caused by the modification of the key toxin domain responsible for binding to the target channel.

## Conclusion

The created construct, based on a short bombesin peptide and a toxin with inhibitor cystine knot (knottin), possesses increased stability simultaneously preserving the ability to bind to cancer cells. This allows us to consider the strategy of creating similar knottin-based structures as a stabilizing scaffold for short peptides for further development of peptide-receptor therapy.
